# Bilateral Alternating Auditory Stimulations Facilitate Fear Extinction and Retrieval

**DOI:** 10.3389/fpsyg.2017.00990

**Published:** 2017-06-14

**Authors:** Sarah Boukezzi, Catarina Silva, Bruno Nazarian, Pierre-François Rousseau, Eric Guedj, Camila Valenzuela-Moguillansky, Stéphanie Khalfa

**Affiliations:** ^1^Institut de Neurosciences de la Timone, UMR 7289, Aix Marseille Université and CNRSMarseille, France; ^2^Instituto Universitário de Lisboa (ISCTE-IUL)Lisboa, Portugal; ^3^Service de Psychiatrie, Hôpital d’Instruction des Armées Sainte-AnneToulon, France; ^4^Centre Européen de Recherche en Imagerie MédicaleMarseille, France; ^5^Assistance Publiques des Hôpitaux de Marseille, Service Central de Biophysique et Médecine NucléaireMarseille, France; ^6^Centro de Estudios de Argumentación y Razonamiento, Facultad de Psicología, Universidad Diego PortalesSantiago, Chile

**Keywords:** fear conditioning, fear extinction, EMDR, skin conductance, stress

## Abstract

Disruption of fear conditioning, its extinction and its retrieval are at the core of posttraumatic stress disorder (PTSD). Such deficits, especially fear extinction delay, disappear after alternating bilateral stimulations (BLS) during eye movement desensitization and reprocessing (EMDR) therapy. An animal model of fear recovery, based on auditory cued fear conditioning and extinction learning, recently showed that BLS facilitate fear extinction and fear extinction retrieval. Our goal was to determine if these previous results found in animals can be reproduced in humans. Twenty-two healthy participants took part in a classical fear conditioning, extinction, and extinction recall paradigm. Behavioral responses (fear expectations) as well as psychophysiological measures (skin conductance responses, SCRs) were recorded. The results showed a significant fear expectation decrease during fear extinction with BLS. Additionally, SCR for fear extinction retrieval were significantly lower with BLS. Our results demonstrate the importance of BLS to reduce negative emotions, and provide a successful model to further explore the neural mechanisms underlying the sole BLS effect in the EMDR.

## Introduction

Posttraumatic stress disorder (PTSD) (Kessler et al., 1995; Alonso et al., 2004) is a highly prevalent occurrence in the aftermath of a traumatic event (American Psychiatric Association [APA], 2013). Patients with PTSD exhibit a number of symptoms including re-experiencing the traumatic event (e.g., flashbacks, nightmares), avoidance of places or objects associated with the initial trauma, fear generalization, and hyper-arousal (American Psychiatric Association [APA], 2004). EMDR therapy has proved to be one of the most efficient therapeutic approaches (Foa et al., 2009; World Health Organization, 2013), and is highly recommended by the American Psychiatric Association [APA] (2004).

Eye movement desensitization and reprocessing is an eight-step standardized protocol of cognitive, emotional, and physical assessment associations of actual distress to traumatic scenery. Also included is imaginal exposure during BLS. These are either auditory, visual, or somaesthetic stimuli alternating between the two sides of the body (Servan-Schreiber et al., 2006). The major therapeutic action of EMDR is achieved through the association of the patient’s traumatic memory and the presentation of BLS (Shapiro, 1996) resulting in an extremely fast extinction of emotional responses elicited by the traumatic memory (Shapiro, 2014).

Fear mechanism deficits are thought to be at the core of PTSD (Milad et al., 2006). PTSD’s underlying mechanism research commonly uses a fear conditioning and extinction paradigm in both human (Milad et al., 2006) and animal studies (Wurtz et al., 2016). This paradigm is based on a repetitive association between an aversive, US and a neutral CS, which leads to a conditioned fear response (LeDoux, 2000).

Despite varying results based on the protocols (with and without context during the fear conditioning), this model has received strong support. Studies have shown that PTSD patients had faster fear conditioning and slower fear extinction (Blechert et al., 2007; Wurtz et al., 2016), as well as a reduced extinction retrieval (Milad et al., 2009).

Adding BLS during fear extinction learning has been used as a model mimicking the core feature of EMDR therapy. It has alleviated early extinction and long-lasting fear recovery of conditioned fear in mice (Wurtz et al., 2016) thus suggesting that fear extinction associated with BLS could constitute a relevant animal model of mechanisms involved in EMDR therapy.

Nevertheless, whether or not a BLS facilitation effect on fear extinction could also be found in humans has not been demonstrated. To further verify if the BLS effect obtained in mice can be replicated in humans, we evaluated 22 healthy participants using a paradigm that included fear conditioning and fear extinction on the first day, and fear extinction recall the following day. We recorded the participants’ behavioral and psychophysiological responses to assess their fear expectations and these responses were recorded by means of skin conductance measurements which have the advantage of reflecting sympathetic nervous system activity, as well as its involved fear response brain structure modulations such as by the amygdala (Knight et al., 2005).

## Materials and Methods

### Participants

Twenty-two healthy participants with no neurological or psychiatric disorders were recruited via screening lists at the *Institut de Neurosciences de la Timone* and *the Faculté de Médecine de la Timone*. Four were excluded because they did not understand the task or they had no conditioning reactions (no difference between the conditioned stimulus CS+ [followed by an electrical US] and the CS- [not associated with the US]). Thus, a total of 18 participants (13 females) with a mean age = 28.4 ± 4.4 years, a mean educational level = 13.4 ± 1.6 years were included. Subject number was determined according to previous studies on fear conditioning and extinction (Milad et al., 2007, 2009).

The investigation was carried out in accordance with the latest version of the Declaration of Helsinki. Participants provided written informed consent in agreement with local ethical committee guidelines set forth by the South Mediterranean 2 Committee who approved the protocol.

### Fear Conditioning, Extinction, and Extinction Recall

**FIGURE 1:**
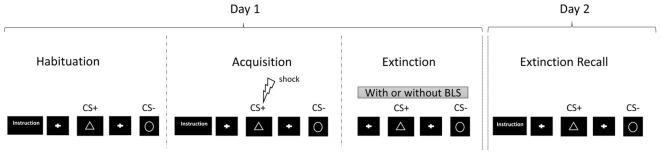
Example that illustrates the experimental protocol including habituation, fear acquisition (= fear conditioning), extinction, and fear extinction recall with two conditions during the extinction phase, *with* and *without* BLS.

The experimental protocol was administrated over two separate days in keeping with the example of previous studies (Milad et al., 2007, 2009). Two fear-conditioning tasks were performed by the participants in a pseudo-randomized order, one *with* and another *without BLS* as illustrated in **Figure 1**.

The BLS were administered using auditory tones alternately delivered to the right and left ears at a frequency of 1 Hz using Sony’s MDRZX110 headset. Auditory tones had broadband sounds similar to those used by many EMDR practitioners with the Tac/AudioScan from NeuroTek Corporation.

On day one, participants underwent the habituation, acquisition, and extinction phases of the two tasks in order to obtain their fear conditioned responses. The BLS were administered during one of the two extinction phases in a pseudo-randomized order. On day two, participants underwent the Recall phases to test for fear extinction retrieval.

Participants were comfortably seated 60 cm from a 17′′ computer screen. In one task, the stimuli were geometric figures of a triangle and a circle, both gray-colored and with identical luminance against a dark-blue background (Merz et al., 2012; Spoormaker et al., 2012). The conditioned stimulus paired with the electric shock (US) was the triangle (CS that was +), and the one not paired was the circle (CS-).

For the other task, the conditioned stimuli were represented by two volumes; a cube and a cylinder, each gray-colored and with identical luminance against a dark-red background. In this case, the CS+ was the cylinder and the CS- was the cube. On each task, the CS+ and the CS- pictures were presented for 4s. The mean inter-trial interval was 8 s (ranging from 6 to 10 s).

On day one, the conditioning task consisted of three different phases: habituation, acquisition, and extinction. The US was a 500-ms electrical stimulation. Electrical stimulation intensity was individually pre-set using an up-down staircase method to achieve a level of “unpleasant but not painful” sensations. The electrical stimulation intensity was kept at a constant all along the conditioning phase.

*The habituation phase* then started with written instructions announcing that two geometric figures would appear on the screen and no shock would be delivered. The habituation phase consisted of 12 trials including six to-be-CS+ and six to-be-CS-.

*During the acquisition phase*, written instructions specified that that two pictures would be shown on the screen, with only one occasionally followed by an electric shock. The CS+ was paired with the US at a partial reinforcement rate of 62, 5%. This phase consisted of 16 CS+ and 16 CS-.

After viewing each image, participants used a 5-button keypad to respond as quickly as possible to: “how much do you expect this stimulus to be paired with an electrical stimulation: 0, 25, 50, 75, or 100%?”

For the extinction phase, no further instructions were shown. This phase consisted of 16 CS+ and 16 CS-. The CS+ was uncoupled with the US.

One of the two extinction phases was associated with auditory BLS. The two tasks and the BLS were presented in a counterbalanced order for all participants and each performed the two conditioning tasks.

On day two, participants performed the extinction recall task related to the two previous conditioning tasks. Participants were presented with the 24 CS+ and the 24 CS- as defined from day one (*with* BLS), and then with the 24 CS+ and 24 CS- (*without* BLS). The two extinction recall tasks were performed successively in counterbalanced order. After each image, the participants, using the 5-button keypad, answered as quickly as possible: “how much do you expect this stimulus to be paired with an electric stimulation: 0, 25, 50, 75, or 100 %?” The CS+ was uncoupled to the US. No BLS were delivered during this phase.

In addition to behavioral responses, SCRs were recorded during all sessions with the Biopac system (MP30). This procedure obtained objective phasic psychophysiological indices of the participant’ sympathetic responses (Boucsein et al., 2012). The SCR were obtained by using two 5 mm inner diameter Ag/AgCl standard electrodes filled with isotonic paste and placed on the left ankle 2-cm from one another in order to reproduce this experiment by using fMRI. This necessitated recording the SCR from electrodes placed as far as possible from the head and the radiofrequency coil. The SCR amplitudes were considered for analysis when they occurred within a 1–3 s latency window after a picture onset and when they were at least equal to 0.01 μS (Boucsein et al., 2012).

### Electrical Stimulation

We used an electric stimulator, developed in partnership with the *Ecole Centrale de Marseille.* This stimulator is powered by a 12 V battery and driven by a digital TTL square command optically isolated. Its TTL pulse is managed by a digital I/O card (NI-6289) under the LabVIEW 2014 (r) environment to load an electronic component. The loaded power depended on the square width (limited to 400 V/0.1 mA) and was discharged to the participant when the square signal returned to 0, using a BIOPAC EL350 plastic bar of two electrodes with concave tin plated disks. Setting a cyclic ratio of a pulse train allows controlling the frequency and the intensity of the 500-ms transcutaneous electrical stimulation. Electrodes were attached to the participants’ left ankle and filled with isotonic past.

### Statistical Analyses

Fear expectation mean percentages and SCR amplitudes were calculated by averaging four subsequent consecutive values resulting from the CS+ and CS- conditions. The averaged four responses, along the conditioning, extinction and recall phases provided nine mean values for each CS-type: one corresponding to habituation (H; the four last), four to conditioning (C1, C2, C3, and C4) and four to extinction (E1, E2, E3, and E4). Results obtained for CS- were subtracted to those for CS+.

Because the data did not follow the normality law and/or did not respect the variance equality, non-parametric tests were performed using sigma plot software (v12). Five participants had no electrodermal conditioning, that is, they had no SCR larger than 0.01 for any of the CS during acquisition. Therefore, our subsequent analyses included the 13 remaining participants with SCR.

To test for the main effect of Time, Friedman repeated the measures ANOVA on ranks on the difference between CS+ and CS- for the fear expectation values (percentages) and the SCR amplitudes of the nine time periods. Tukey’s HSD tests were used for *post hoc* comparisons when appropriate. The Wilcoxon signed rank test was conducted to test for effect of BLS between the *“with BLS”* condition and the *“without BLS”* condition, for each of the nine time periods.

## Results

### Fear Conditioning and Extinction

**FIGURE 2:**
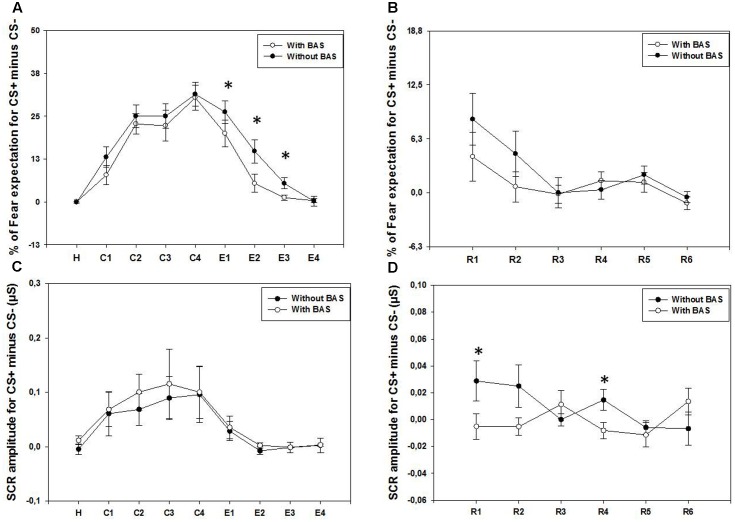
**(A,C)** The results during habituation (H), fear conditioning (C1, C2, C3, and C4), and fear extinction (E1, E2, E3, and E4). **(A,B)** The means and standard error bars of percentage of fear expectation for the difference between CS+ and CS–. These are displayed for both conditions, *with* and *without* BLS. **(C,D)** The means and standard error bars for the SCRs for the difference between CS+ and CS– for the two conditions are presented. **(B,D)** Results for the fear extinction retrieval (R1, R2, R3, R4, R5, and R6). As for the figures **(A,C)** during fear condition and extinction, means and standard errors of CS+ minus CS– are displayed for fear expectation **(B)** and SCRs **(D)**, in both conditions. ^∗^Indicate significant (*p* < 0.05) difference between the two BLS conditions.

As displayed on the **Figure 2A**, the results showed a significant effect of Time for the percentage of fear expectation for the difference between CS+ and CS-, *with BLS* (χ^2^ = 72.1, *p* < 0.001), and *without BLS* (χ^2^ = 78.6, *p* < 0.001). *Post hoc* comparisons in both conditions showed a significantly larger fear expectation for C2, C3, and C4 than for the habituation and the extinction (E2, E3, and E4) periods (*p* < 0.05), with the exception of *without BLS*, where no significant differences between C4 and E2 were found. This result is in accordance with the fact that fear expectation during the extinction is lower *with BLS* compared to *without BLS* at E1, E2, and E3 (respectively, *W* = 79, *p* < 0.05; *W* = 114.3, *p* < 0.005; *W* = 28, *p* < 0.05). However, this faster decrease of fear expectation during extinction was not found for the SCR amplitudes (**Figure 2C**). In addition, a significant Time effect on the SCR was only *with BLS* (χ^2^ = 30.2, *p* ≤ 0.001), showing larger amplitudes for C2 than for the habituation and E3 (*p* < 0.05). No significant effect of Time was found *without BLS* (χ^2^ = 9.3, *p* > 0.05).

### Fear Extinction Retrieval

As displayed on the **Figure 2B**, there is a significant effect of Time during the fear extinction recall for the percentage of fear expectation, both *with* (χ^2^ = 17.8, *p* < 0.005) and *without BLS* (χ^2^ = 21.01, *p* < 0.001) conditions. For the two conditions, fear expectations at the onset of the recall (R1), were greater than at the end of the recall (R6, *p* < 0.05). Contrary to the extinction period, during fear extinction retrieval, no significant effect of BLS was found, regardless of the time period (*p* > 0.05). Conversely, for the SCR amplitudes (**Figure 2D**), the difference between CS+ and CS- did not change over Time *with BLS* (χ^2^ = 6.3, *p* > 0.05) and *without BLS* (χ^2^ = 8.7, *p* > 0.05). Contrary to the fear expectation results, SCR amplitudes for the difference between CS+ and CS- were larger during the extinction recall (R1 and R4) *without BLS*, as compared to *with BLS* (respectively, *W* = 67, *p* < 0.05; *W* = 24, *p* < 0.05).

## Discussion

The present study findings replicate in humans the fear extinction facilitation by BLS previously found in mice (Wurtz et al., 2016). Even if the fear responses during fear extinction recall were not sensitive to BLS when assessed by the participants, their physiological responses showed a response modulated by BLS, as it did in mice. Thus, the current results confirm that BLS decreases fear responses during both fear extinction and fear extinction recall in humans which suggests that results in animals can be translated to humans.

The BLS effect was found during extinction for the percentage of fear expectation but not during fear extinction recall. Conversely, the BLS effect exists during fear extinction recall but not during extinction for SCR indicating a dissociation between the behavioral and the psychophysiological responses. This lack of BLS effect on the SCRs during the fear extinction may be related to habituation of the electrodermal measure (Steiner and Barry, 2011). Habituation refers to a decline in response amplitude and probability as a function of stimulus repetition (Siddle, 1991). Since the extinction occurred after the presentation of 44 visual stimuli, SCRs were therefore strongly susceptible to habituation, masking a possible BLS impact.

The lack of BLS effect on fear expectation during the fear extinction retrieval could be due to the weak fear expectation difference between CS+ and CS- (<10% on average) suggesting that participants minimally expected fear.

Further experiments could improve the protocol of fear extinction recall to avoid such a ceiling effect. This could be achieved by enhancing the fear expectations during the fear conditioning, then increasing the duration of the conditioning phase and reinforcement rate of the US to elicit larger fear expectation responses at the recall. Nevertheless, the fact that SCR amplitudes were larger *without BLS* compared to *with BLS* during fear extinction recall seems to confirm that fear had been felt even, if it was reduced, compared to fear conditioning. This may be the case, since the SCRs are known to be modulated by the brain structure underlying the fear response, that is, the amygdala (Knight et al., 2005).

The significant Time effect found on the percentage of fear expectation substantiates the validity of our fear conditioning protocol. In particular, this effect of Time showed an increased fear expectation until the end of the fear conditioning, and a decrease along the fear extinction for the difference between CS+ and CS-. There are similar observations for the fear extinction recall since the fear expectation for the CS+ minus CS- was higher at the beginning than at the end of the fear extinction recall.

This Time effect was less evident for the SCR amplitudes and could be due to two reasons. Firstly, the statistical power for the SCR results was weaker when compared to that of the behavioral responses. This is because the number of participants included in the SCR’s analyses was reduced when compared to the behavioral sample (five of the participants did not show any apparent SCRs to the stimuli). Moreover, according to the standard errors, it seems that there was greater variability of the SCR than of the behavioral responses.

Secondly, as reported above, the SCRs are potentially susceptible to habituation (Steiner and Barry, 2011). The fact that the protocol was completed twice by each participant (*with* and *without* BLS) could have elicited a greater habituation in the electrodermal responses, and might have occurred from the beginning of the fear extinction recall phase. Nonetheless, the conditioning effect on SCR was significant (*with* BLS), and the curves followed the same tendency as those found for the fear expectation (**Figure 1**).

As with mice (Wurtz et al., 2016), current BLS effect parallels what happened during EMDR therapy. That is, a decrease of the emotions related to a trauma event represented here by the fear of the electric shock memory. The BLS during the fear extinction paradigm seems to trigger a biological reaction reflected by a decrease of the SCR during the fear extinction recall, as well as a decrease of fear expectation during fear extinction.

Given that the SCR are modulated by the amygdala (Knight et al., 2005) and that fear perception notably involves the thalamo-amygdala and anterior cingulate cortex interactions (Das et al., 2005), it is plausible that the BLS may act on the fear responses through an action on these structures. This hypothesis is worth verifying in future MRI studies and could clarify whether such a mechanism could be at the core of the EMDR therapy.

In agreement with this hypothesis, recent reviews argue that BLS may be essential to the EMDR therapy (Jeffries and Davis, 2013). Moreover, a functional MRI study also showed enhanced amygdala activity in participants while viewing pictures representing disgust, as compared to neutral ones, in the presence of alternating tones (Herkt et al., 2014). Our protocol could therefore serve to further explore and understand the neurophysiological mechanisms underlying the BLS effect during EMDR therapy, a question that, to date, remains unanswered (Jeffries and Davis, 2013).

Some authors theorize that the EMDR effect mostly relies upon the taxing memory effect of eye movements (van den Hout et al., 2011, 2014; Leer et al., 2014) while others suggest that the EMDR allows memory reconsolidation (Oren and Solomon, 2012).

In our experiment, the BLS effect during fear extinction may rely on such a taxation of vividness and emotionality of the electric stimulation memory, or may provoke memory reconsolidation. Our model may serve to explore and solve this issue considering the sole effect of the BLS.

Future studies should also further consider variables such as age and gender that could be potential limitations to the interpretation of the BLS effect.

This experiment demonstrated the facilitating effect of BLS on fear extinction learning and its retrieval. Further experiments using the present protocol coupled with functional MRI, and applying the refinements suggested above, would be effective to explore the brain mechanisms involved in the “BLS effect,” and likely in the EMDR, to decrease negative emotions related to a traumatic memory. In addition, further experiments could also verify, as in Wurtz et al. (2016), that alternating stimuli is really more effective than unilateral or non-alternating stimulations as in the Servan-Schreiber et al. (2006) study.

## Author Contributions

SK, SB, CS, BN, P-FR, EG, and CV-M substantially contributed to the design and to the experiment and the acquisition, analysis and the interpretation of the data, and to drafting and final approval of the version of the manuscript to be published. The agreement is accountable for all aspects of the work in ensuring that questions related to the accuracy or integrity of any part of the work are appropriately investigated and resolved.

## Conflict of Interest Statement

The authors declare that the research was conducted in the absence of any commercial or financial relationships that could be construed as a potential conflict of interest.

## Abbreviations

BLS: bilateral alternating stimulations
CS: conditioned stimulus
EMDR: eye movement desensitization and reprocessing
SCR: skin conductance responses
US: unconditioned stimulus.
